# GBP2 promotes podocyte pyroptosis and contributes to the pathogenesis of pediatric lupus nephritis

**DOI:** 10.1371/journal.pone.0344601

**Published:** 2026-03-19

**Authors:** Yu Sun, Jiahui Wei, Yanjun Zhao, Xingbo Jiang, Chao He, Xianhui Huang, Fengying Lei, Yuanhan Qin

**Affiliations:** Department of Pediatrics, The First Affiliated Hospital of Guangxi Medical University/Guangxi Clinical Research Center for Pediatric Diseases (Focuses on Complex and Critical Cases), Nanning, China; Novartis Institutes for BioMedical Research, UNITED STATES OF AMERICA

## Abstract

**Background:**

Lupus nephritis (LN), a prevalent and serious manifestation of systemic lupus erythematosus, exhibits particularly high incidence in children, yet its pathogenesis remains incompletely elucidated. This study aimed to investigate differentially expressed genes in LN and elucidate their regulatory mechanisms.

**Methods:**

Bioinformatics analysis was conducted on the publicly available glomerular gene expression dataset GSE32591 from the Gene Expression Omnibus to identify hub genes. Based on this screening, Guanylate-binding protein 2 (*GBP2*) was selected for further investigation. In vivo, GBP2 expression was assessed in renal tissues from pediatric LN patients by immunohistochemistry. In a murine LN model, the expression levels of GBP2 and key pyroptosis-associated markers were evaluated using immunohistochemistry, RT-qPCR, and western blotting. In vitro, a podocyte pyroptosis model was induced by lipopolysaccharide and adenosine triphosphate treatment. Functional experiments involving *Gbp2* knockdown and overexpression, followed by a rescue experiment where absent in melanoma 2 (*Aim2*) was overexpressed in *Gbp2*-knockdown cells, were performed to elucidate its role and underlying mechanisms in regulating pyroptosis.

**Results:**

Bioinformatics analysis identified *OAS1*, *OAS2*, *IRF7*, *GBP2*, and *GBP1* as hub genes. An upregulation of GBP2 and gasdermin D (GSDMD) was observed in the glomeruli of children with LN, showing a strong correlation with 24-hour urinary protein excretion. Renal tissues from LN mice exhibited markedly increased expression of GBP2, AIM2, Caspase-1, and GSDMD compared to controls. Following siRNA-mediated knockdown of *Gbp2* in vitro, a consequent reduction was observed in pyroptosis-associated proteins (GSDMD, AIM2) and diminished secretion of the pro-inflammatory cytokines IL-1β and IL-18. Conversely, *Gbp2* overexpression aggravated these effects. Pyroptosis suppressed by *Gbp2* knockdown was partially restored upon concurrent overexpression of *Aim2*.

**Conclusion:**

Our results demonstrate that GBP2 expression is significantly upregulated in LN and promotes podocyte pyroptosis, likely contributing to renal injury. These findings suggest that GBP2 facilitates the progression of pediatric LN by activating the pyroptotic pathway and triggering the release of inflammatory cytokines.

## Introduction

Lupus nephritis (LN), a serious complication of systemic lupus erythematosus (SLE), frequently manifests clinically as nephrotic syndrome characterized by varying degrees of proteinuria, renal impairment, and potentially renal failure [1,2]. Approximately 50%−80% of SLE patients develop kidney damage during the disease course, with roughly 22% progressing to renal insufficiency [3–5]. LN is more prevalent and severe in pediatric-onset SLE compared to adults [4]. These children face substantial morbidity due to the chronic relapsing disease course and prolonged immunosuppressive therapy, which adversely impacts growth and development [6,7]. The signs and symptoms of LN do not always reflect the severity of the disease, and current treatment strategies struggle to achieve sustained complete renal remission rates [8,9]. Therefore, exploring LN pathogenesis and developing novel effective therapies are crucial for advancing clinical practice.

In recent years, bioinformatics studies based on genome-wide association studies have identified multiple biological pathways involving differentially expressed genes (DEGs) in LN, primarily associated with pathogen infection, inflammation, interferon signaling pathways, NOD-like receptor activation, and immune responses [10–13]. Notably, Guanylate-binding protein 2 (*GBP2*) emerges as a core gene in LN pathogenesis, with immunohistochemical studies confirming its elevated expression in renal tissues of adult LN patients [14]. GBP2, a member of the interferon-inducible GBP family, plays a key role in host defense by regulating inflammasome activation and pyroptosis, particularly in response to intracellular bacterial pathogens [15]. Pyroptosis, a lytic and inflammatory type of programmed cell death, drives the initiation and progression of renal diseases [16]. Building on this evidence, our study analyzed a public LN dataset and also identified *GBP2* as a candidate DEG. A comprehensive investigation into the interplay between GBP2 and pyroptosis is crucial for a complete understanding of LN pathogenesis.

In this study, we employed bioinformatics methods to analyze genetic data from LN and normal control kidney tissues to identify disease-associated DEGs. We validated GBP2 expression in pediatric LN patients and murine LN kidneys, and functionally characterized its regulatory role in pyroptosis through in vitro experimentation. These results reveal novel aspects of LN pathogenesis, thereby informing the development of targeted therapeutic strategies.

## Materials and methods

### Data acquisition and analysis

The gene expression data GSE32591 was obtained from the Gene Expression Omnibus (GEO) database (http://www.ncbi.nlm.nih.gov/geo) on 01/03/2024. We extracted the glomerular tissue data, which included glomerular tissue samples from 29 LN patients and 32 normal controls.

The limma package (v3.44.3) in R was used to identify DEGs, applying the thresholds of |log₂FC| > 1 and adjusted *P*-value < 0.05. Genes with log₂FC > 1 were defined as upregulated, whereas those with log₂FC <−1 were considered downregulated.

Gene Ontology (GO) biological processes and Kyoto Encyclopedia of Genes and Genomes (KEGG) pathway analyses were performed on upregulated DEGs using the DAVID database (https://david.ncifcrf.gov/tools.jsp). Data visualization was performed using the online platform (https://www.bioinformatics.com.cn).

For the subsequent protein-protein interaction (PPI) network analysis, we focused on the “NOD-like receptor signaling” pathway, which was significantly enriched. We selected all upregulated DEGs (n = 13) annotated to this pathway. These genes were imported into the STRING database (https://string-db.org) employing a minimum interaction confidence threshold of 0.4. The resulting network was visualized and analyzed in Cytoscape (v3.10.33), with hub genes identified using the Maximal Clique Centrality (MCC) algorithm via the CytoHubba plugin.

### Patient cohort

This study retrospectively collected laboratory results and renal biopsy specimens from 56 pediatric patients hospitalized at the First Affiliated Hospital of Guangxi Medical University between 01/01/2013 and 31/12/2023. The cohort consisted of 50 patients with active LN (40 females and 10 males; age range 5-17 years) and 6 patients who underwent nephrectomy for renal tumors. The latter serving as a source of non-inflammatory renal tissue controls. The 50 LN patients were randomly selected from those who met the American College of Rheumatology classification criteria for SLE, had biopsy-confirmed LN, and had no evidence of genetic, metabolic, or other autoimmune disorders. The control group comprised 6 individuals who underwent nephrectomy due to renal tumors, providing histologically normal renal tissue for comparison.

Renal tissue samples, fixed in formalin and embedded in paraffin, were retrospectively collected from the Department of Pathology, the First Affiliated Hospital of Guangxi Medical University. The laboratory results included 24-hour urinary protein (24hUP) levels measured within the 72 h period preceding renal biopsy. Data were accessed for research purposes on 01/04/2024. During patient recruitment and data collection, investigators had access to personally identifiable information to enable linkage between clinical records and laboratory samples. All identifiable data were maintained under strict confidentiality and stored separately from the analytical dataset. Direct personal identifiers were removed immediately after data collection, and analysis was performed using anonymized codes. The key linking these codes to identities was securely stored and accessible only to the principal investigator. Researchers had no access to participant identities during or after the study. Ethical approval for this study was obtained from the Ethics Committee of the First Affiliated Hospital of Guangxi Medical University (Approval No. 2024-K034-01). Informed consent was obtained from the legal guardians of all participants.

### Animal models and tissue harvesting

Six 9-week-old female MRL/lpr mice were utilised as the LN model (Changzhou Cavens Experimental Animal Co., Ltd), and six age-matched female C57BL/6J mice were used as healthy controls (Guangzhou Vital River Laboratory Animal Technology Co., Ltd.). All mice were maintained in a specific pathogen-free environment within the Guangxi Medical University animal facility. A standardized photoperiod of 12 h of light and 12 h of darkness was maintained, while food and water were provided without restriction.

Random urine samples were collected weekly using metabolic cages following an acclimatization period. We anesthetized the mice at 21 weeks of age with an intraperitoneal injection of pentobarbital sodium (50 mg/kg). Retro-orbital bleeding was performed for blood collection, followed by bilateral nephrectomy.

All experimental protocols were approved by the Animal Ethics Committee of Guangxi Medical University (Approval No. 202402016) and conducted in accordance with the NIH Guide for the Care and Use of Laboratory Animals.

### Cell culture and treatment

Mouse Podocyte Clone-5 cells (MPC-5; Cyagen Biosciences, Guangzhou, China; M2-0701) were maintained in DMEM supplemented (Gibco) with 10% fetal bovine serum (FBS) (Sigma-Aldrich, St. Louis, MO, USA; F0193) at 37°C in 5% CO_2_. To simulate the inflammatory microenvironment relevant to LN, we employed a well-established in vitro model involving the sequential combination of LPS as a priming signal and ATP as an activation signal to induce pyroptosis. Its applicability to podocytes is supported by prior studies [17–19]. MPC-5 cells were stimulated with LPS (1 μg/mL; Sigma-Aldrich; L4391) for 24 h to provide the priming signal, followed by ATP (5 mM; Sigma-Aldrich; A6419) for 2 h to induce inflammatory stress and pyroptosis-related responses. Gene modulation was achieved using *Gbp2*-targeting siRNA (GeneCreate, Wuhan, China) and pEGFP-N1-*Gbp2* and pEGFP-N1-*Aim2* overexpression plasmids (Shenggong Bioengineering, Shanghai, China). Before LPS treatment, cells were transiently transfected using Lipofectamine 8000 transfection reagent (Beyotime Biotechnology, Shanghai, China; C0533) following the manufacturer’s instructions. Cells were divided into ten experimental groups: (1) Untreated control; (2) LPS/ATP (LA); (3) LPS/ATP + negative control siRNA (si-NC); (4) LPS/ATP + *Gbp2*-targeting siRNA (si-GBP2); (5) LPS/ATP + negative control plasmid (ov-NC); (6) LPS/ATP + *Gbp2* overexpression plasmid (ov-GBP2); (7) LPS/ATP + *Aim2* overexpression plasmid (ov-AIM2); (8) LPS/ATP + si-NC + ov-NC (dual-transfection negative control); (9) LPS/ATP + si-GBP2 + ov-NC; (10) LPS/ATP + si-GBP2 + ov-AIM2. For each experimental condition, a minimum of three technical replicates were used, and all experiments were independently repeated at least three times with cells from different passages to ensure biological reproducibility.

### Cell viability and membrane integrity assays

Podocyte viability and pyroptosis‑associated plasma membrane damage were assessed using the Cell Counting Kit‑8 (CCK-8) (UElandy Inc., Suzhou, China; C6005L) and a Lactate Dehydrogenase (LDH) Release Assay Kit (Beyotime Biotechnology; C0016), respectively. For the CCK‑8 assay, 10 μL of reagent was added per well post‑stimulation; after 2 h incubation at 37°C, absorbance was measured at 450 nm. For the LDH assay, supernatants were collected post‑stimulation, incubated with the working solution for 30 min in the dark, and absorbance was read at 490 nm after stopping the reaction. LDH release (%) was normalized to the maximum release obtained from cells lysed with the kit’s lysis reagent. All experiments included a minimum of three independent biological replicates.

### RNA extraction and quantitative real-time PCR (RT-qPCR)

Following extraction with FreeZol Reagent (Vazyme Biotech, Nanjing, China; R711) from murine renal tissues and MPC-5 cells, total RNA was reverse-transcribed into cDNA using the PrimeScript RT Master Mix (Vazyme; R323). RT-qPCR was performed using ChamQ Universal SYBR qPCR Master Mix (Vazyme; Q711) under the following thermal profile: initial denaturation at 95°C for 30 seconds; 40 cycles of denaturation at 95°C for 10 seconds and combined annealing/extension at 60°C for 30 seconds; concluding with melt curve analysis. Following amplification with sequence-specific primers (custom-synthesized by Shenggong Bioengineering; sequences are listed in Table 1) and normalization to glyceraldehyde-3-phosphate dehydrogenase (GAPDH), relative expression was quantified via the 2^−ΔΔCt^ method using technical triplicates.

**Table 1:** Primers used in current study.

Gene	Forward Primer Sequence (5’-3’)	Reverse Primer Sequence (5’-3’)
Gbp2	AGCAGCACCTTCATCTACAACAGC	CACCTCCATTGTCCCTGTTTTAT
Aim2	GTCACCAGTTCCTCAGTTGTG	CACCTCCATTGTCCCTGTTTCAT
Caspase-1	AGAGGATTGCTTAACGGATGCA	GCACACGACCAGGCATATTCTT
Gsdmd	GATCAAGGAGGTAAGCGGCA	CGACTCCGGTTCTGGTTCTGG
Gapdh	AGGTCGGTGTGAACGGATTTG	TGTAGACCATGTAGTTGAGGTCA

### Western blot assay (WB)

Protein extraction was performed on murine renal tissues and MPC-5 cells. The RIPA lysis buffer (Solarbio Life Sciences, Beijing, China; R0010) was supplemented with 1% protease inhibitor to prevent degradation prior to extraction. Lysates were centrifuged, and supernatants were collected and protein concentration was determined using a BCA assay kit (Thermo Fisher Scientific, Waltham, MA, USA; 23227). The samples were then resolved on 10% SDS-PAGE gels and electrotransferred onto PVDF membranes (Merck Millipore, Burlington, MA, USA). The membranes were blocked for 2 h at room temperature with 5% skimmed milk (Beyotime) in TBST, followed by an overnight incubation at 4 °C with primary antibody against GBP2 (Immunoway Biotechnology, Suzhou, China; YN1744; 1:1000), AIM2 (Immunoway Biotechnology;YM8846; 1:2000), Caspase-1 (Immunoway Biotechnology;YM9369, 1:4000), GSDMD (Nature Biosciences, Beijing, China; A22998; 1:2000), and GAPDH (Share-Bio, Shanghai, China; SB-AB2000; 1:10000) as loading control. Membranes were washed three times for 10 minutes each in TBST and incubated for 1 h at room temperature with an HRP-conjugated goat anti-rabbit secondary antibody (Proteintech Group, Rosemont, IL, USA; SA00001-2) diluted at 1:10,000. Protein bands were visualized using HRP chemiluminescent substrate (Biosharp Life Sciences, Hefei, China; BL520A) and quantified using ImageJ software (v1.54g).

### Immunohistochemistry staining

Immunohistochemical staining was performed on paraffin-embedded renal sections to verify core gene expression in LN. Sections underwent deparaffinization in xylene, rehydration through graded alcohols, and heat-mediated antigen retrieval in citrate buffer (pH 6.0). Following blocking of endogenous peroxidases with 3% H_2_O_2_ and non-specific binding sites with 10% normal serum, sections were incubated at 4°C overnight with primary antibodies targeting GBP2 (Proteintech; 27299-1-AP; 1:400) and GSDMD (Proteintech; 20770-1-AP; 1:400), followed by 1 h room temperature incubation with HRP-conjugated secondary antibody (AiFang Biological, Changsha, China; AFIHC003). Sections were visualized using diaminobenzidine (DAB) (AiFang Biological; AFIHC004) and counterstained with hematoxylin. Digital images were acquired using a Nikon microscope. Five randomly selected high-power fields (400×) per sample were quantified using ImageJ software (v1.54g). Glomerular and tubular regions were identified and quantified separately based on morphological landmarks. Only fields containing intact glomeruli were selected for glomerular quantification. After converting images to 8-bit and applying a consistent threshold to identify DAB positivity, the integrated optical density (IOD) of the positive signal was measured. The average IOD from the five fields per sample was used for statistical analysis.

### Enzyme-linked immunosorbent assay (ELISA)

Following the manufacturer’s protocol, the concentrations of double-stranded DNA (dsDNA) (Cusabio Technology, Wuhan, China; CSB-E11194m) in mouse serum and the levels of IL-1β (ELK Biotechnology, Wuhan, China; ELK1271) and IL-18 (ELK Biotechnology; ELK2269) in the culture supernatant of MPC-5 cells were quantified using ELISA.

### Measurements of urinary protein

Renal function in mice was assessed by measuring urinary protein (Nanjing Jiancheng Bioengineering Institute, Nanjing, China; C035-2-1), urine creatinine (Nanjing Jiancheng; C011-2-1), and blood urea nitrogen (Nanjing Jiancheng; C013-2-1) with commercial kits, according to the manufacturer’s instructions.

### Statistical analysis

All data were analyzed using GraphPad Prism 7.0 software and graphs expressed as means ± SD of at least three independent experiments. The two-tailed Student’s t-test was used to make between-group comparisons. One-way ANOVA followed by Tukey’s post hoc test was applied for multi-group comparisons. Correlation analyses for data not conforming to a normal distribution were performed using Spearman’s rank correlation. A *P* < 0.05 was considered statistically significant.

## Results

### Screening hub genes

**Fig 1:**
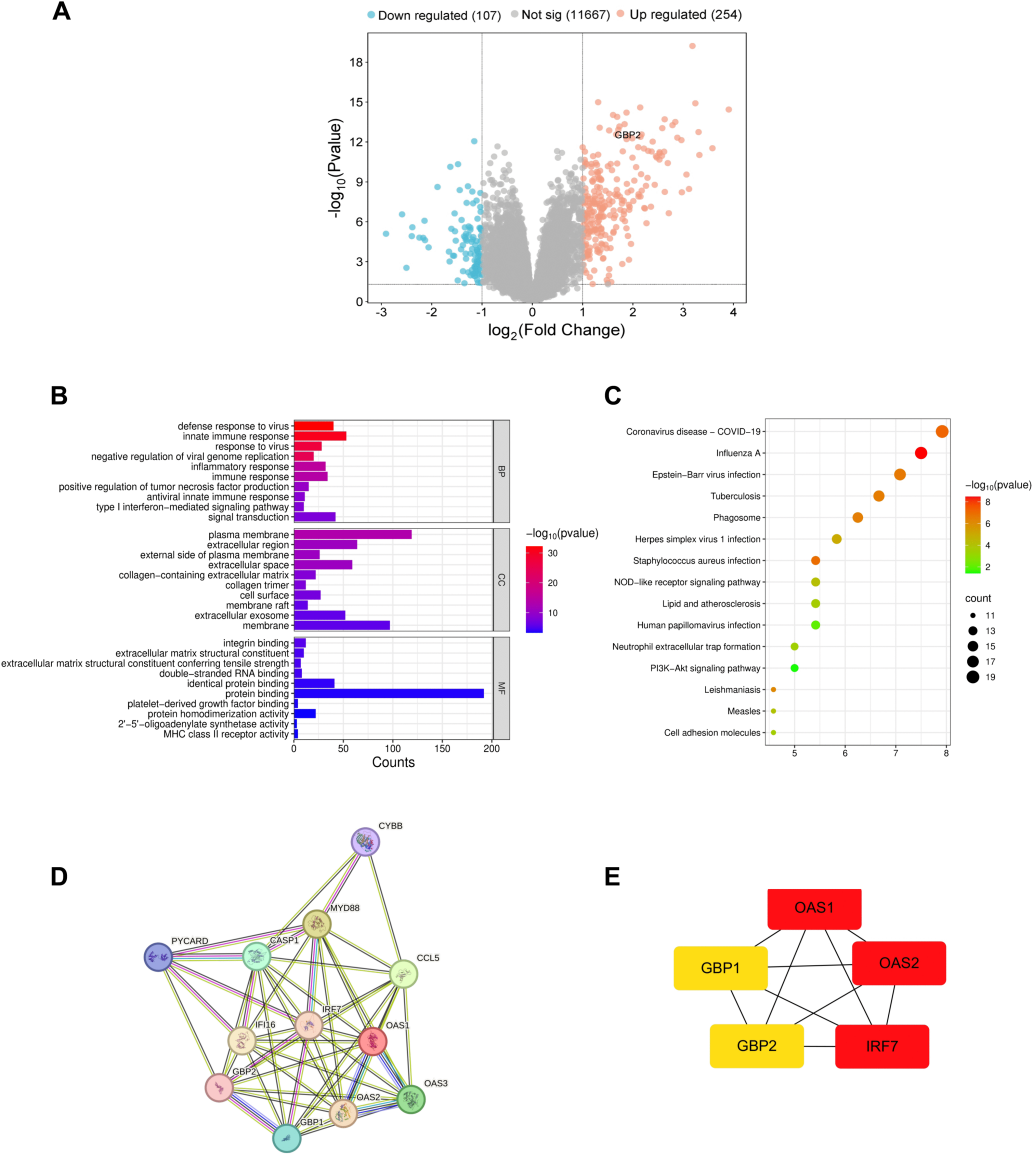
Functional enrichment analysis of differentially expressed genes (DEGs) in lupus nephritis. **A** Volcano plot of DEGs in LN and normal control samples. Blue dots represent significantly downregulated DEGs in the samples, and red dots represent significantly upregulated DEGs. **B** GO functional enrichment analysis was performed on the upregulated DEGs, and the top 10 of biological process (BP), cell component (CC), and molecular function (MF) terms were selected to draw a bar graph. **C** Functional enrichment of upregulated DEGs analyzed by DAVID, with the top 15 pathways shown by bubble plot. **D** 13 DEGs enriched in the NOD-like receptor signaling pathway were imported into the STRING database, and the protein interaction network was processed with Cytoscape. **E** The top 5 hub DEGs were obtained by using the MCC algorithm of the Cytohubba plug-in in Cytoscape software.

Bioinformatic analysis identified 361 DEGs in LN glomeruli compared to controls, with 254 upregulated and 107 downregulated transcripts (Fig 1A).

GO enrichment of DEGs was classified according to biological process (BP), cellular component (CC), and molecular function (MF). For upregulated DEGs, they are significantly enriched in defense response to virus, innate immune response, and response to virus in BP, plasma membrane, extracellular region, external side of plasma membrane in CC, and integrin binding, extracellular matrix structural constituent, and extracellular matrix structural constituent conferring tensile strength in MF (Fig 1B).

Enrichment analysis of KEGG pathways revealed that the top 15 upregulated DEGs ranked by ascending *P*-value were enriched in coronavirus disease (COVID-19), influenza A virus, EB virus infection, tuberculosis, phagocytosis, herpes simplex virus 1 infection, Staphylococcus aureus infection, alongside the NOD-like receptor signaling pathway (Fig 1C). Among the 15 KEGG pathways ranked by gene count, the NOD-like receptor pathway ranked eighth overall and represented the top non-pathogen-specific mechanism. PPI analysis of the 13 DEGs enriched in the NOD-like receptor signaling pathway via STRING and Cytoscape revealed *OAS1*, *OAS2*, *IRF7*, *GBP2*, and *GBP1* as the top five hub genes identified through the MCC algorithm (Fig 1D and 1E).

### GBP2 and GSDMD are highly expressed in LN and associated with proteinuria

**Fig 2:**
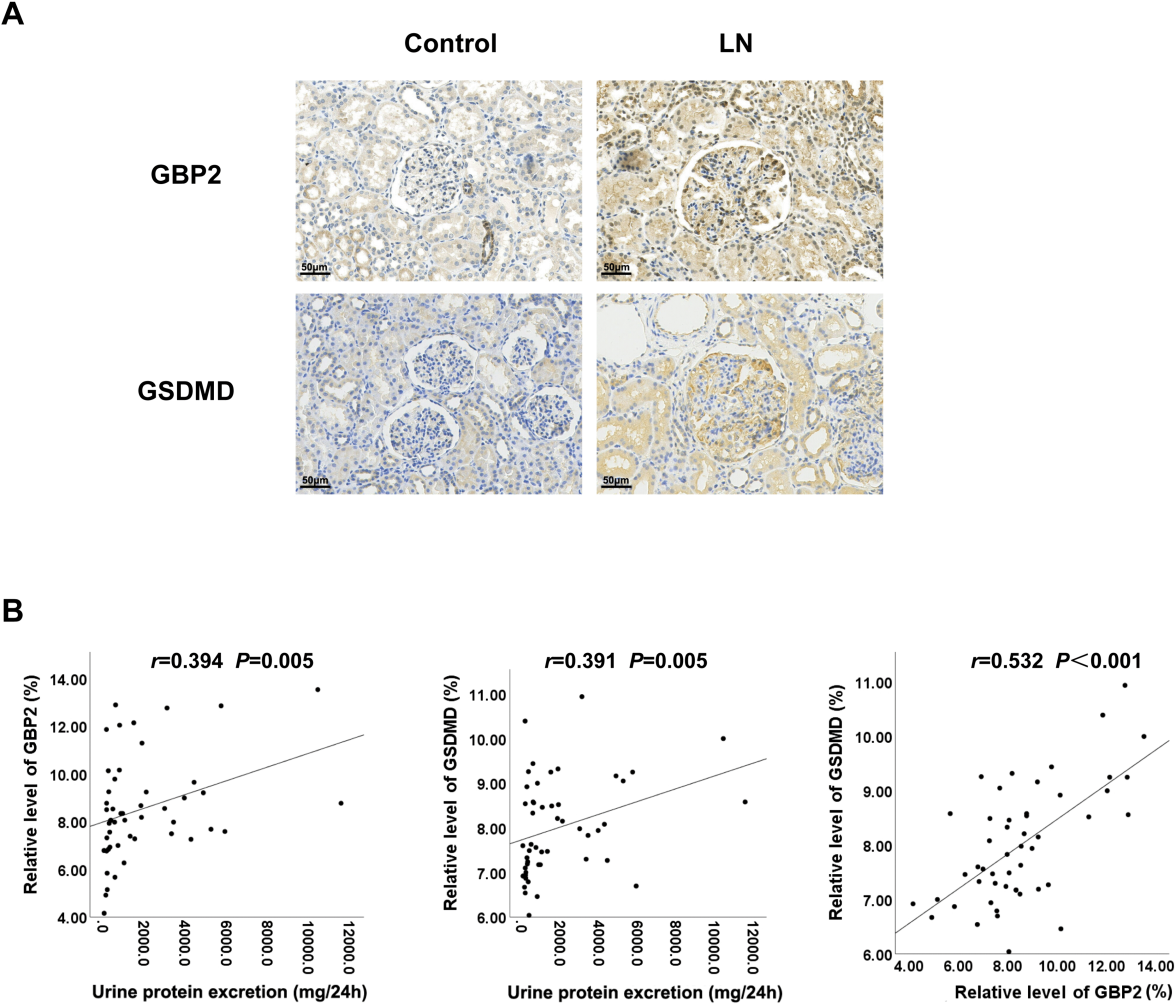
Immunohistochemical analysis of GBP2 and GSDMD in pediatric lupus nephritis. **A** Representative immunohistochemical images (400 × magnification; scale bar, 50 μm) of renal sections from pediatric Lupus nephritis (LN) and control renal tissues, showing the expression and localization of GBP2 and GSDMD in glomeruli and tubules. **B** Glomerular areas were manually outlined in five randomly selected 400 × fields for mean optical density quantification. Correlation analyses of glomerular GBP2 or GSDMD expression with proteinuria in pediatric LN patients (n = 50) were shown, with statistical significance determined by Spearman correlation.

The age distribution and gender ratio of patients with LN and the control group are summarized in Table 2. Immunohistochemical analysis confirmed significantly elevated GBP2 and GSDMD expression in pediatric LN renal tissues compared to controls (Fig 2A). Control specimens exhibited minimal expression restricted to tubular epithelium, whereas LN tissues demonstrated robust immunopositivity across multiple renal compartments, including glomerular mesangium, endothelial cells, podocytes, and tubular epithelium.

**Table 2:** Age and gender distribution in the lupus nephritis and control groups.

Characteristic	Lupus Nephritis Group (n = 50)	Control Group (n = 6)	*P* value
Age (years)			
Median [IQR]	12 [11, 13]	11.5 [11, 12]	0.698
Gender, n (%)			
Male	10 (20)	1 (16.7)	>0.999
Female	40 (80)	5 (83.3)	

Quantitative assessment revealed significant positive correlations between glomerular GBP2 expression and 24hUP levels (Spearman’s *r* = 0.394, *P* = 0.005), glomerular GSDMD expression and 24hUP levels (Spearman’s *r* = 0.391, *P* = 0.005), as well as intrarenal GBP2 and GSDMD protein levels (Spearman’s *r* = 0.532, *P* < 0.001) (Fig 2B).

### GBP2 and pyroptosis-associated proteins were highly expressed in LN mouse renal tissues

**Fig 3:**
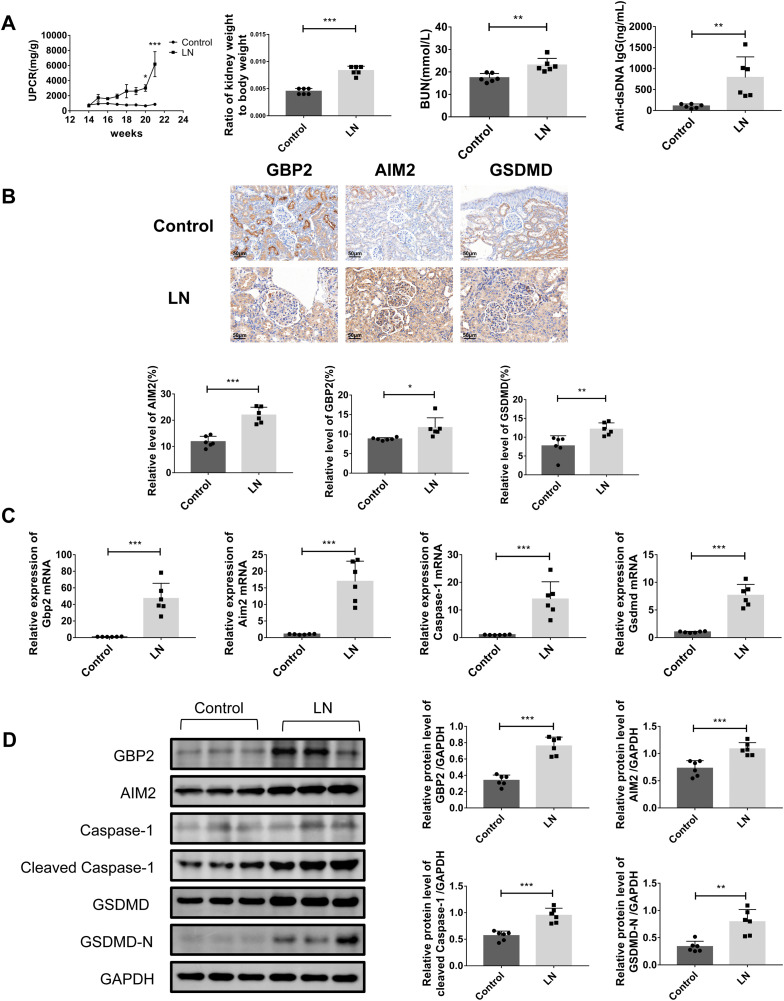
Characterization of renal injury, GBP2 expression, and pyroptosis in LN mice. **A** Evaluation of urinary protein-creatinine ratio, kidney-to-body weight ratio, blood urea nitrogen and anti-dsDNA antibody levels in control and Lupus nephritis (LN) mice. **B** Representative immunohistochemical staining of renal tissues in control and LN mice (400 × magnification; scale bar, 50 μm). **C** Evaluation of pyroptosis-related mRNA levels by RT-qPCR. **D** Evaluation of pyroptosis-related protein levels by western blotting. Data were expressed as the mean ± SD (n = 6 per group). *P < 0.05, **P < 0.01, ***P < 0.001.

The LN mouse model demonstrated characteristic disease progression, with MRL/lpr mice exhibiting significantly elevated urinary protein-to-creatinine ratios (UPCR) at week 21 compared with C57BL/6J controls, alongside marked nephromegaly evidenced by increased kidney-to-body weight ratios. Disease group mice also showed significantly elevated serum biomarkers, including blood urea nitrogen (BUN) and anti-dsDNA antibodies (Fig 3A).

Immunohistochemical analysis of control specimens displayed limited GBP2, AIM2 and GSDMD immunopositivity primarily in tubules, whereas kidneys from LN group mice exhibited robust expression of GBP2, AIM2, and GSDMD throughout glomerular and tubular compartments (Fig 3B). RT-qPCR and WB confirmed significant upregulation of pyroptosis pathway components in renal tissues, with GBP2, AIM2, Caspase-1, and GSDMD expression levels all substantially elevated in diseased versus control mice (Fig 3C and 3D).

### Silencing *Gbp2* significantly inhibits LPS/ATP-induced podocyte pyroptosis

**Fig 4:**
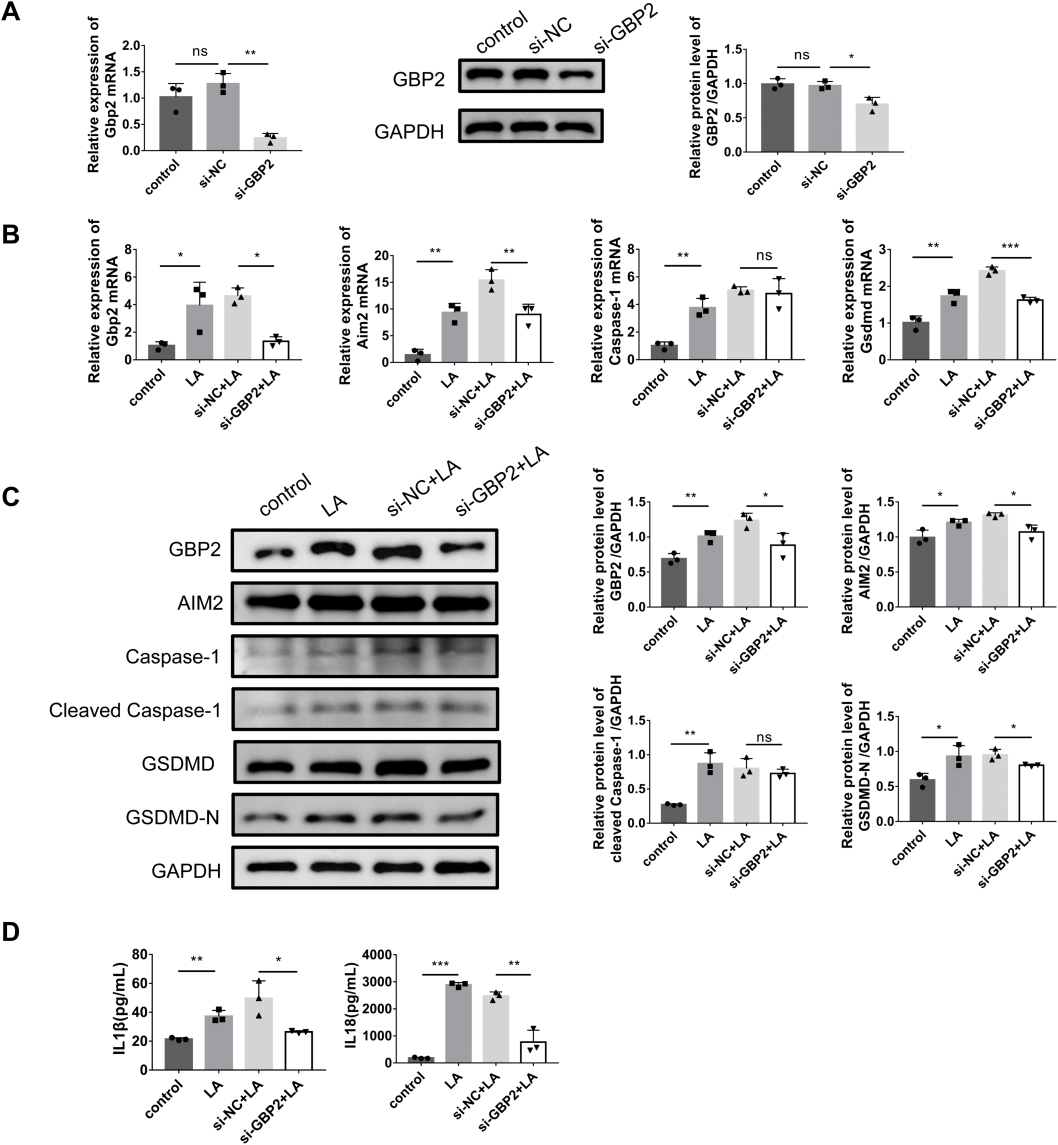
Effects of *Gbp2* silencing on pyroptosis and inflammation in MPC-5 cells. **A** Evaluation of siRNA knockdown efficiency by RT-qPCR and western blotting. **B** Evaluation of pyroptosis-related mRNA levels by RT-qPCR. **C** Evaluation of pyroptosis-related protein levels by western blotting. **D** Evaluation of inflammatory **cytokines** by ELISA. Data were expressed as the mean ± SD (n = 3 independent biological replicates). *P < 0.05, **P < 0.01, ***P < 0.001; ns, not significant.

The sequential combination of LPS as a priming signal and ATP as an activation signal is a well-established and standardized in vitro model for inducing canonical NLRP3 inflammasome activation and subsequent pyroptosis. Its specific applicability to renal parenchymal cells, including podocytes in the context of LN is supported by prior mechanistic studies [17–19].To investigate the functional role of GBP2 in LN, we performed *Gbp2* knockdown and examined its effects on LPS/ATP-stimulated MPC-5 cells. After 24 h siRNA transfection, cell samples were collected for RT-qPCR analysis, and proteins were collected at 48 h post-transfection for WB. Post-transfection analyses revealed significantly reduced GBP2 expression in si-GBP2 group versus siRNA-negative control (si-NC) cells at both mRNA and protein levels (Fig 4A).

We first confirmed that the LPS/ATP model recapitulated the disease-relevant upregulation of GBP2. In the LPS/ATP group (LA), GBP2 mRNA and protein levels were significantly increased relative to the control group (Fig 4B and 4C), consistent with observations in LN patient and murine tissues.

Within this validated model, we next examined whether GBP2 regulates pyroptosis. As expected, LPS/ATP stimulation markedly upregulated key pyroptosis-executing molecules (GSDMD, Caspase-1, AIM2). Importantly, *Gbp2* silencing suppressed LPS/ATP-induced GSDMD and AIM2 expression relative to si-NC group (Fig 4B and 4C). Furthermore, while LPS/ATP potently elevated IL-1β and IL-18 levels, the addition of si-GBP2 significantly inhibited these inflammatory responses, and si-NC had no effects on them (Fig 4D). In summary, pyroptosis and the overexpression of inflammatory cytokines induced by LPS/ATP were significantly inhibited following *Gbp2* silencing.

### *Gbp2* overexpression significantly promotes LPS/ATP-induced podocyte pyroptosis

**Fig 5:**
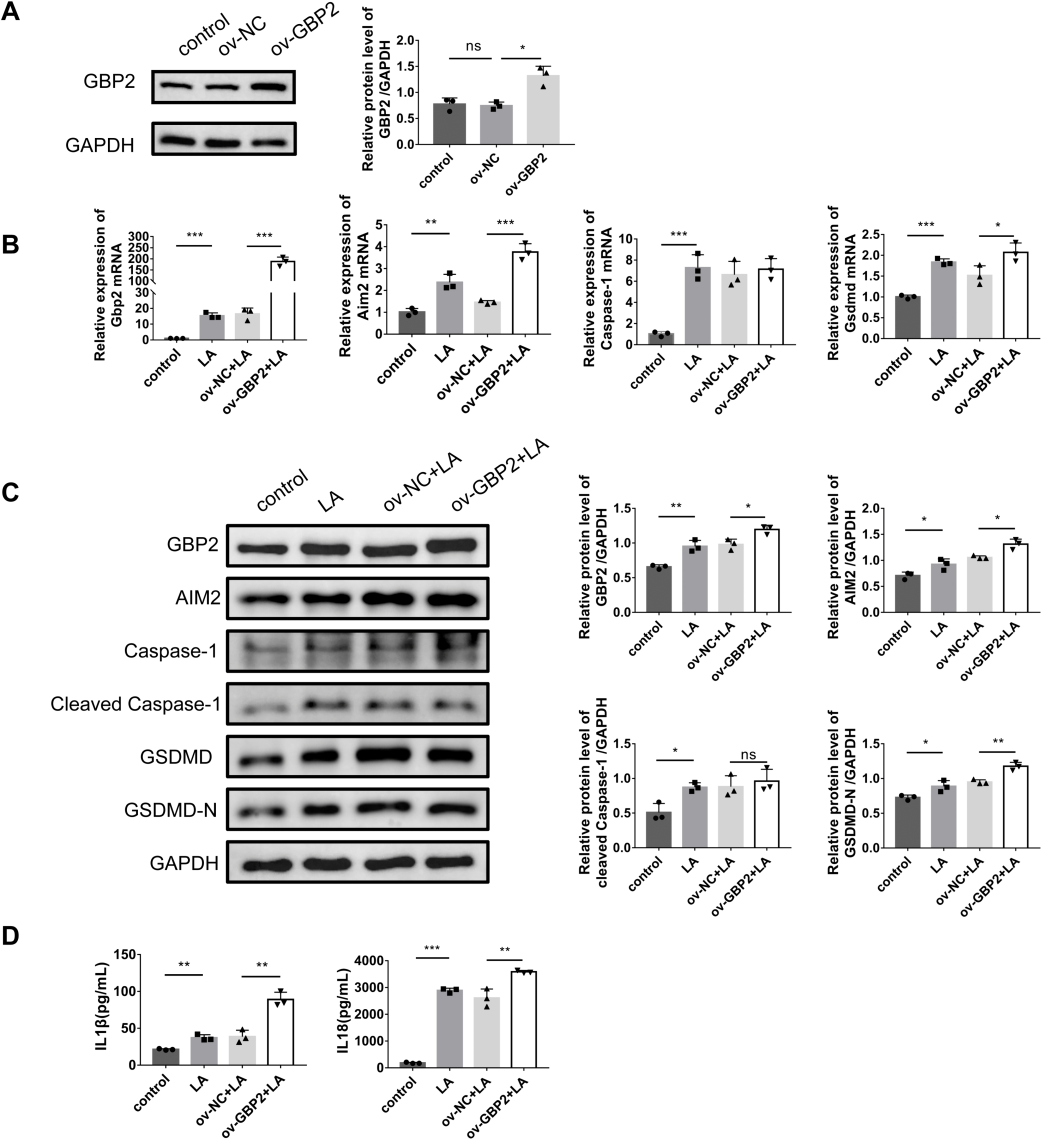
Effects of *Gbp2* overexpression on LPS/ATP-induced pyroptosis and inflammation in MPC-5 cells. **A** Evaluation of plasmid overexpression efficiency by RT-qPCR and western blotting. **B** Evaluation of pyroptosis-related mRNA levels by RT-qPCR. **C** Evaluation of pyroptosis-related protein levels by western blotting. **D** Evaluation of inflammatory **cytokines** by ELISA. Data were expressed as the mean ± SD (n = 3 independent biological replicates). *P < 0.05, **P < 0.01, ***P < 0.001; ns, not significant.

In the opposite way, to further investigate GBP2’s role in LPS/ATP-induced MPC-5 cells, we performed *Gbp2* overexpression using pEGFP-*Gbp2* transfection, confirming significant upregulation at the protein level (Fig 5A). *Gbp2* overexpression significantly upregulated mRNA and protein levels of GSDMD and AIM2 compared to ov-NC group (Fig 5B and 5C). Concomitantly, the inflammatory cytokines IL-1β and IL-18 showed a significant increase (Fig 5D). Collectively, these findings demonstrate that *Gbp2* overexpression promotes pyroptosis and inflammatory responses.

### *Aim2* overexpression partially rescues the pyroptosis phenotype attenuated by *Gbp2* knockdown

**Fig 6:**
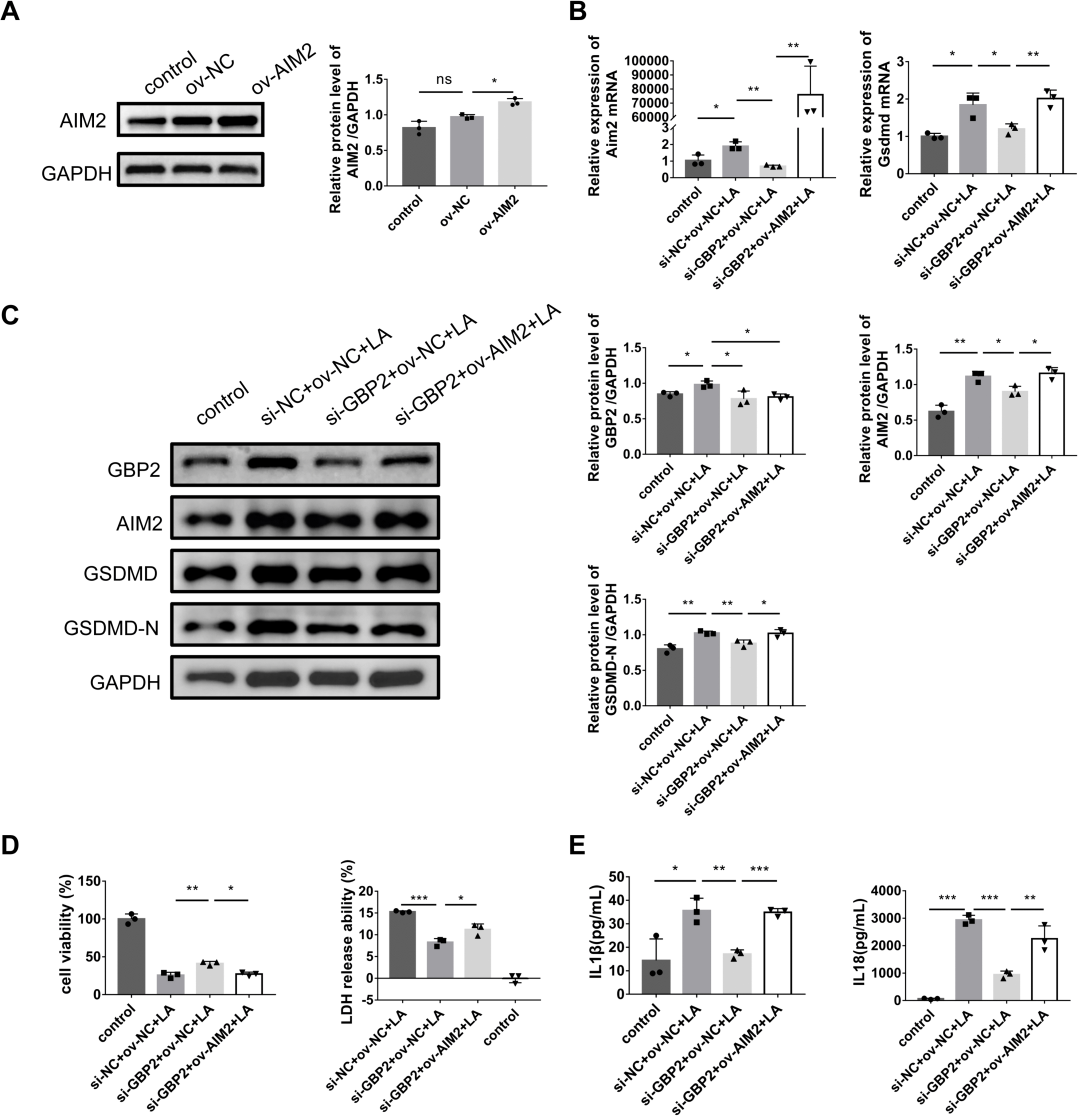
*Aim2* overexpression rescues the inhibitory effects of *Gbp2* knockdown on pyroptosis in MPC-5 cells. **A** Evaluation of plasmid overexpression efficiency by western blotting. **B** Evaluation of pyroptosis-related mRNA levels by RT-qPCR. **C** Evaluation of pyroptosis-related protein levels by western blotting. **D** Cell viability (assessed by CCK-8 assay) and plasma membrane damage (assessed by LDH release assay) across treatment groups. **E** Evaluation of inflammatory **cytokines** by ELISA. Data were expressed as the mean ± SD (n = 3 independent biological replicates). *P < 0.05, **P < 0.01, ***P < 0.001; ns, not significant.

To determine whether AIM2 is a functional downstream mediator of GBP2, a rescue experiment was performed. We performed *Aim2* overexpression using pEGFP-*Aim2* transfection, confirming significant upregulation at protein levels (Fig 6A). Then MPC-5 cells were co-transfected with si-GBP2 and ov-AIM2. Notably, *Aim2* overexpression partially rescued the suppression of GSDMD expression (Fig 6B and 6C) caused by *Gbp2* silencing alone. Consistent with these molecular changes, the **increase** in cell viability (assessed by CCK-8 assay) and the decrease in lactate dehydrogenase (LDH) release, both induced by *Gbp2* knockdown, were also partially reversed upon *Aim2* co-overexpression (Fig 6D). Similarly, the inhibition of IL-1β and IL-18 secretion was partially restored (Fig 6E). Collectively, these results indicate that *Aim2* overexpression can partially reverse the anti-pyroptotic effects of *Gbp2* knockdown, supporting the conclusion that GBP2 promotes pyroptosis in podocytes, at least in part, through regulating AIM2.

## Discussion

SLE is a chronic autoimmune disorder characterized by multi-system inflammation. Approximately 70% of SLE patients develop LN [20,21]. The pathogenesis of LN remains complex and incompletely elucidated. Studies have found that the pathogenesis of SLE is related to the overactivation of immune pathways related to antiviral response [22,23].

This study conducted differential expression and enrichment analysis on whole-genome microarray expression matrices derived from glomerular samples of LN patients and normal controls obtained from the GEO database. We constructed a PPI network centered on 13 upregulated DEGs enriched in the NOD-like receptor signaling pathway and identified the top 5 hub DEGs: *OAS1*, *OAS2*, *IRF7*, *GBP2*, and *GBP1*. Both *GBP1* and *GBP2* belong to the *GBP* family. GBPs are interferon-inducible proteins, typically exhibiting low basal expression in tissues such as the liver, lungs, kidneys, and digestive tract. However, elevated interferon levels rapidly induce GBPs expression, enabling them to play crucial regulatory roles in immune responses against infections, inflammation, and tumors [24]. Accumulating evidence links GBPs to SLE and LN. For instance, elevated levels of GBP1 are detected in the serum of SLE patients, where it acts as a critical mediator governing inflammatory cell pyroptosis [25]. GBP3 shows higher expression in the kidneys of LN mouse models compared to controls. Furthermore, *GBP3* knockout in LPS-treated human glomerular epithelial cells promoted cell proliferation while reducing the expression of key inflammatory cytokines, including IL-1β, TNF-α, and IL-8, as well as proteins associated with pyroptosis, such as GSDMD, Caspase-1, and NLRP3. Conversely, *GBP3* overexpression exerted the opposite effects [26]. Similarly, GBP5 expression was increased in the LN mouse renal cortex. *Gbp5* knockout significantly reduced 24hUP, BUN and creatinine levels, and attenuated kidney damage in LN mice. In vitro, *Gbp5* deletion attenuated inflammation through suppression of NLRP3 inflammasome activation [27]. Here, we evaluated the contribution of GBP2 to the pathogenesis of LN.

GBP2 activates inflammasomes and induces cell pyroptosis by hydrolyzing guanosine triphosphate during intracellular pathogen clearance, releasing LPS and dsDNA [24]. Recent research reveals the critical role of GBP2 in kidney disease progression. In diabetic nephropathy, GBP2 activates Notch1 signaling to promote macrophage M1 polarization, serving as a prognostic biomarker and therapeutic target [28]. Elevated GBP2 expression also correlates with poor prognosis in renal clear cell carcinoma [29]. Studies have shown that GBP2 is a core interferon response gene associated with LN. Furthermore, immunohistochemical analysis has demonstrated that GBP2 expression is significantly elevated in the renal biopsies of adult LN patients relative to those with membranous nephropathy or renal microangiopathy [14]. Consistent with previous studies, we observed significant overexpression of GBP2 and GSDMD in the glomeruli of pediatric LN patients. Proteinuria is closely associated with early remission and prognosis in LN [30,31]. To determine if GBP2 expression is linked to clinical disease severity in LN, we analyzed its correlation with proteinuria. Results showed that both GBP2 and GSDMD protein levels exhibited a significant positive correlation with 24hUP. Additionally, analysis identified a marked positive correlation between the expression levels of GBP2 and GSDMD. Consistent with the human data, renal tissues from LN mice displayed significantly higher levels of both GBP2 and GSDMD compared to normal controls. Collectively, these findings suggest that elevated GBP2 expression may exacerbate renal cell pyroptosis, contributing to glomerular damage and proteinuria in LN. These results indicate that GBP2 could serve as a biomarker for assessing LN severity. Quantitative assessment of renal tissues from murine models demonstrated a marked increase in the expression of AIM2 and Caspase-1 at both protein and transcriptional levels in the LN group relative to control animals. This finding implicates AIM2 activation in LN pathogenesis, prompting us to investigate whether GBP2 exacerbates renal injury specifically through this pathway.

Given the pivotal role of podocytes in maintaining the glomerular filtration barrier and their susceptibility to injury in LN [32–37], we next sought to determine whether GBP2 promotes podocyte pyroptosis via the AIM2 inflammasome. After combined LPS/ATP treatment, mRNA and protein levels of AIM2, Caspase-1, and GSDMD in podocytes significantly increased, while protein levels of IL-1β and IL-18 in cell supernatants also markedly elevated, indicating that this approach induces MPC-5 cell pyroptosis. Following exposure to stimuli that trigger pyroptosis, a substantial increase in both GBP2 mRNA and protein was observed, consistent with a potential functional role for this molecule. To functionally interrogate this relationship, we modulated *Gbp2* expression. Knockdown of *Gbp2* in pyroptosis-induced podocytes resulted in significantly reduced mRNA and protein expression of AIM2 and GSDMD, indicating that *Gbp2* expression intervention can influence the pyroptosis process. Conversely, overexpression of *Gbp2* in podocytes led to significantly elevated mRNA and protein levels of AIM2 and GSDMD. To directly test if AIM2 is a functional downstream effector, we performed a rescue experiment. Notably, co-overexpression of *Aim2* in *Gbp2*-knockdown podocytes partially restored the level of GSDMD and the secretion of IL-1β/IL-18 that were suppressed by *Gbp2* silencing alone. Collectively, our data confirm that GBP2 promotes podocyte pyroptosis by regulating the key downstream effector AIM2, thereby driving podocyte damage in the context of LN.

Notably, while GBP2-mediated upregulation of AIM2 was associated with enhanced pyroptotic outcomes, like GSDMD activation and cytokine release, it did not proportionately increase cleaved Caspase-1. This discrepancy suggests that the canonical Caspase-1-dependent execution may not be the primary route in this context. Alternative executioners, such as Caspase-8, which can be recruited by AIM2 under certain conditions, or a highly transient Caspase-1 activation state, could underlie this phenomenon [38,39]. Further studies are needed to unravel the more detailed mechanism.

This study has certain limitations. First, the control group was small (n = 6) due to the challenge of obtaining normal pediatric renal tissue; samples were thus derived from non-neoplastic regions of tumor nephrectomy specimens. While clinically relevant, the small control size limits statistical power for direct comparisons and precludeds rigorous individual matching for age and sex. Although no significant demographic differences were found, and variables were adjusted for statistically, residual confounding remains possible. Consequently, we focused our core analyses on the larger LN cohort. Therefore, conclusions derived from direct human tissue comparisons should be interpreted with caution and require validation in larger, independent cohorts. Nonetheless, the pronounced upregulation of GBP2 and GSDMD in LN tissues versus controls provides consistent phenotypic support for our key conclusions. Second, we utilized sequential LPS/ATP stimulation to model LN-relevant podocyte injury. While this reductionist approach is suitable for dissecting the specific role of GBP2 within this defined pathway, it does not encompass the full complexity of the LN microenvironment, nor does the use of a murine podocyte line fully recapitulate human podocyte biology in vivo. Therefore, while our findings clarify the mechanism of GBP2 in pyroptosis, the in vivo relevance and translational potential require further validation. Future work should aim to expand the clinical cohort through multicenter collaboration and to confirm the role of GBP2 using LN animal models.

In summary, this study demonstrates that GBP2 and GSDMD are significantly upregulated in renal tissues from pediatric LN patients and exhibit a positive correlation with proteinuria severity, suggesting a potential role in LN-associated renal injury. Using an LPS/ATP-induced pyroptosis model in podocytes, we found that modulating GBP2 expression influences AIM2 inflammasome activation and pyroptosis. However, GBP2 does not appear to regulate podocyte pyroptosis via canonical, full activation of the AIM2 inflammasome, as evidenced by the limited change in cleaved Caspase-1. Given the complex pathogenesis of LN, which involves inflammatory responses, oxidative stress, and metabolic abnormalities, GBP2 may participate in multiple signaling pathways and likely exerts stage-specific mechanisms. Therefore, further investigation is essential to elucidate the mechanistic role of GBP2 in podocyte pyroptosis to develop innovative strategies for LN prevention and treatment.

## Conclusion

This study demonstrated that GBP2 expression was markedly elevated in pediatric LN patients and was associated with increased proteinuria. Experiments conducted in podocyte cultures revealed that GBP2 regulates pyroptosis. Mechanistically, GBP2 appears to prime the pyroptotic response primarily by upregulating the expression of the inflammasome sensor AIM2. These observations imply that targeting GBP2 may offer a novel strategy for treating LN.

## Supporting information

S1 File Raw images. (PDF)

S2 File Original data. (XLSX)
