# Supplementary figures and images for: GBP2 promotes podocyte pyroptosis and contributes to the pathogenesis of pediatric lupus nephritis
Source: PLoS One. 2026 Mar 19;21(3):e0344601. doi: 10.1371/journal.pone.0344601 (PMC13001939; doi:10.1371/journal.pone.0344601)

# Raw Images

Fig 3D

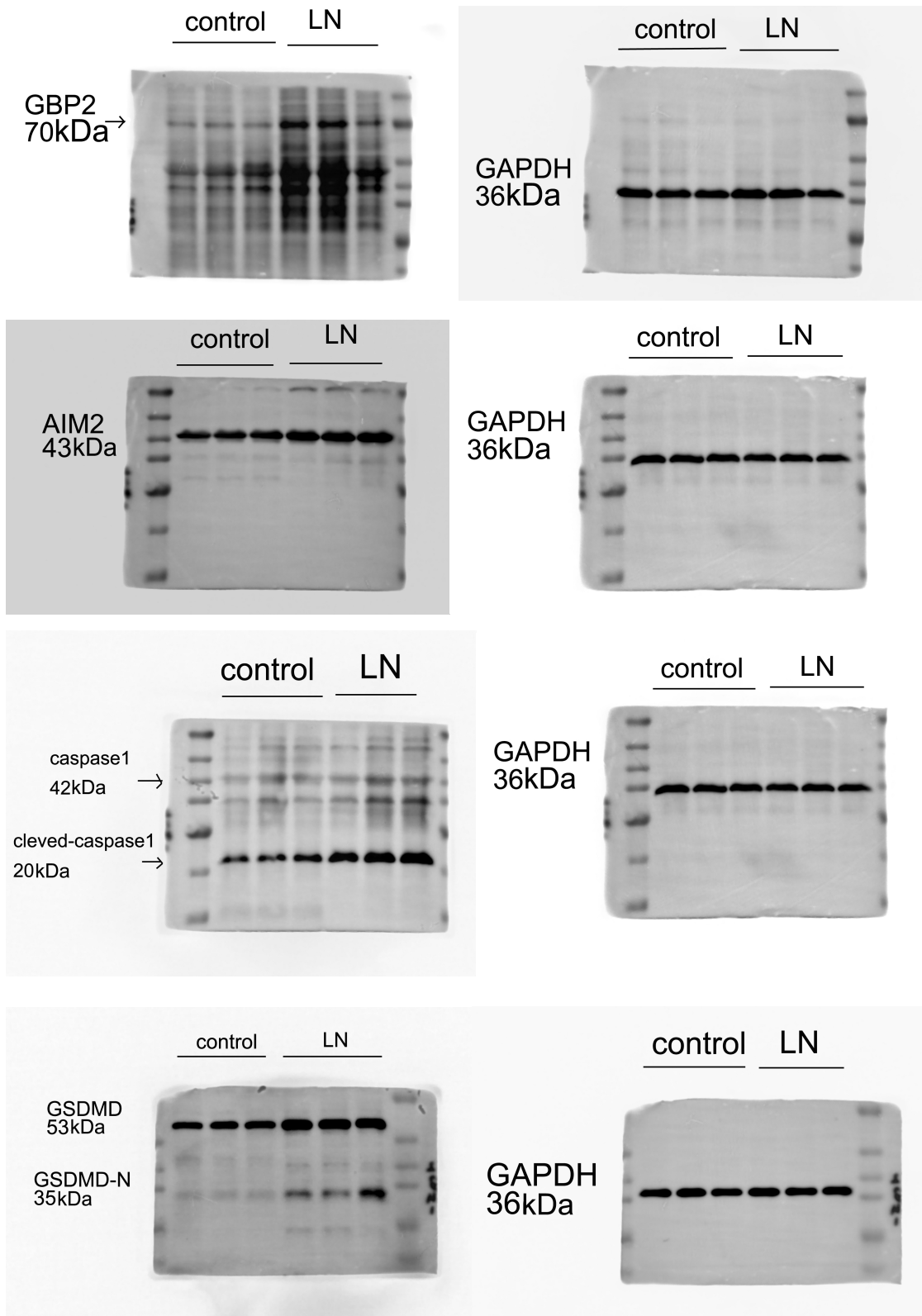

Fig 4A

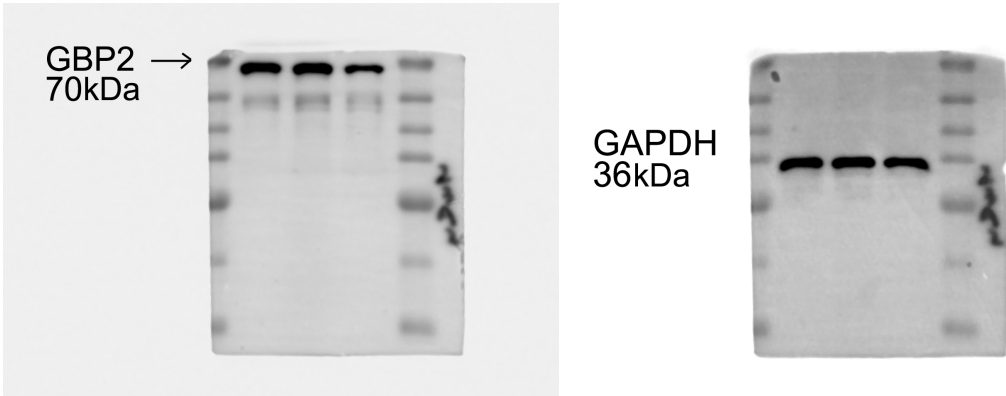

Fig 4C

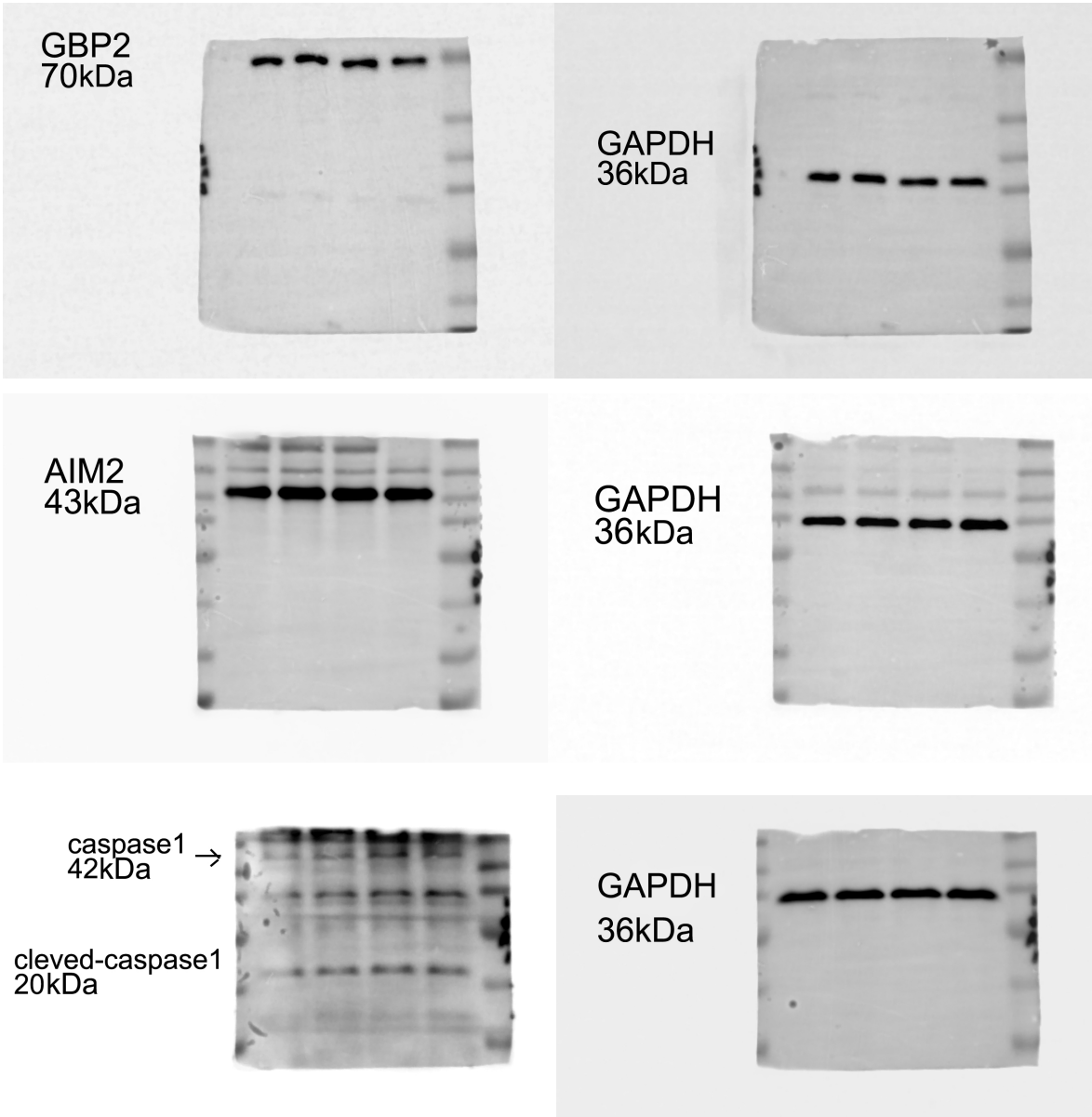

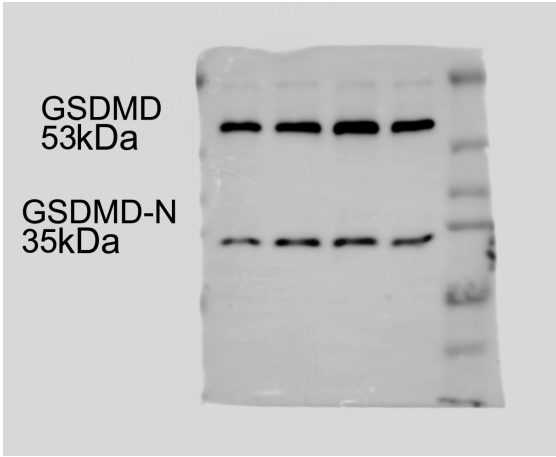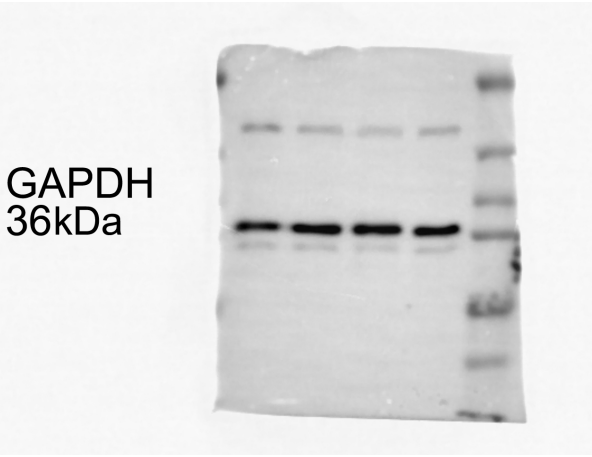

Fig 5A

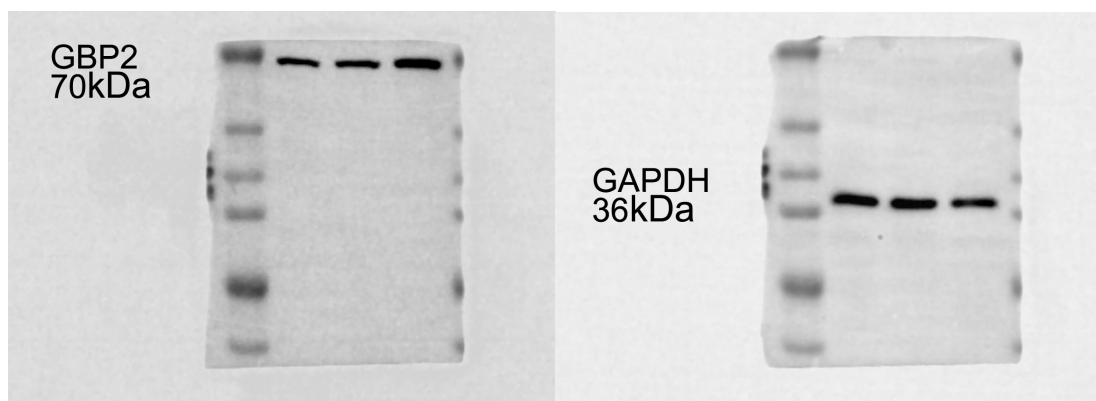

Fig 5C

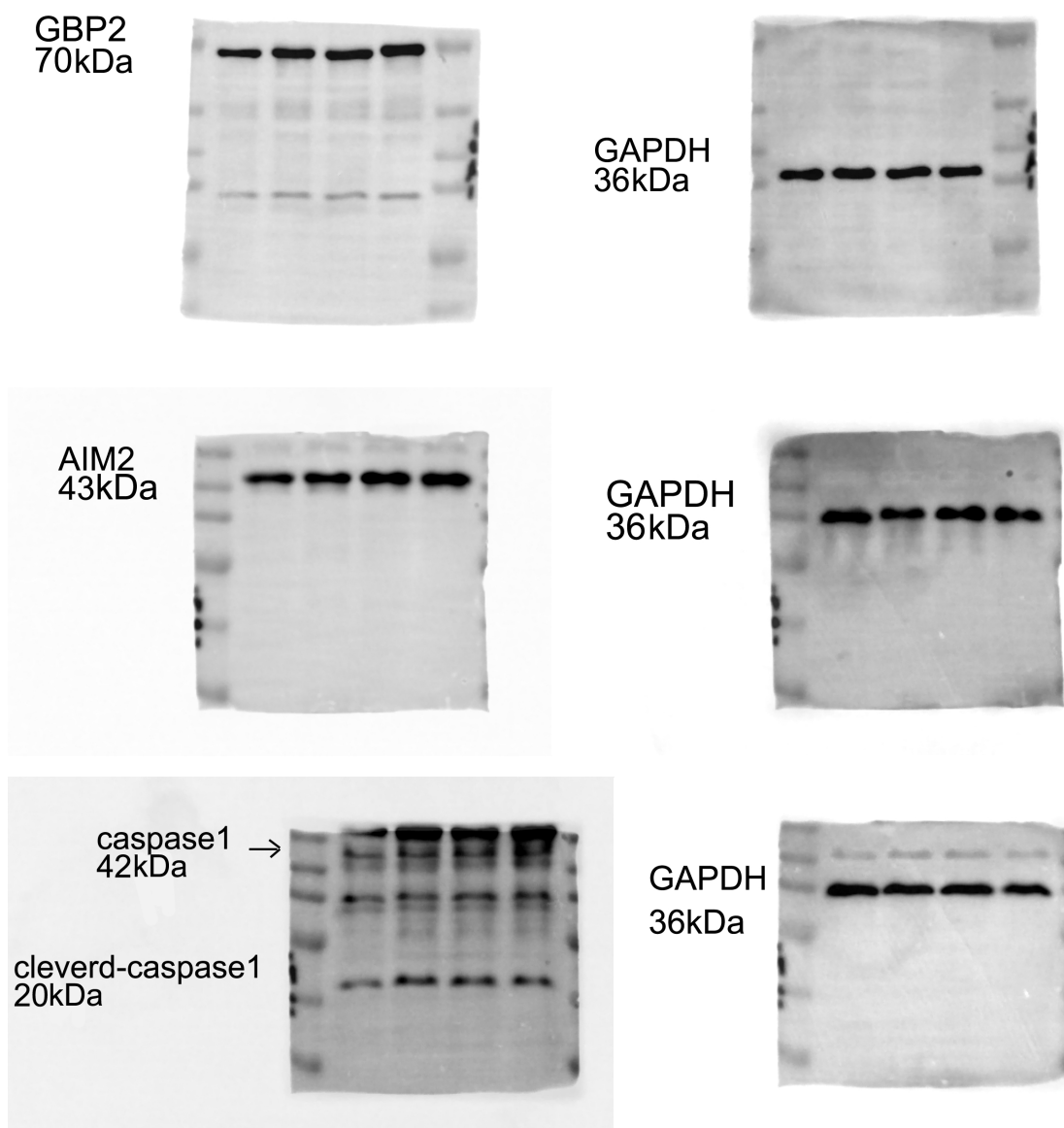

GADMD  
53kDa

GSDMD-N  
35kDa

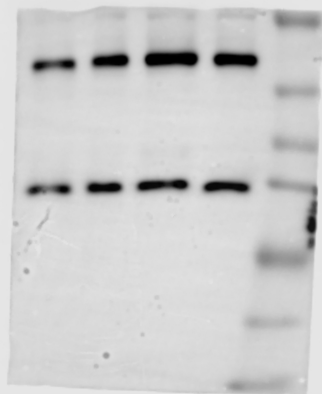

GAPDH  
36kDa

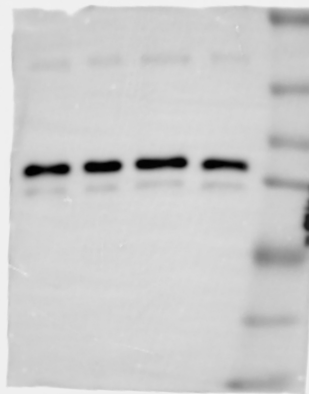

Fig 6A

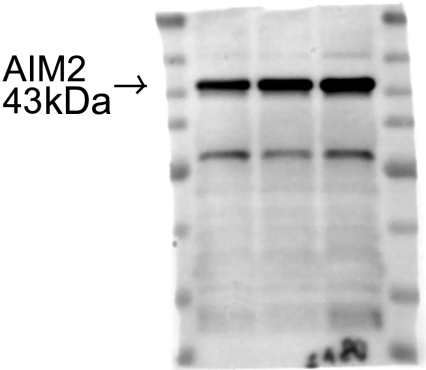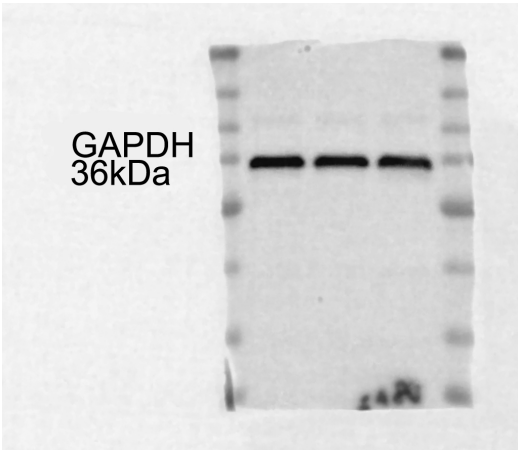

Fig 6C

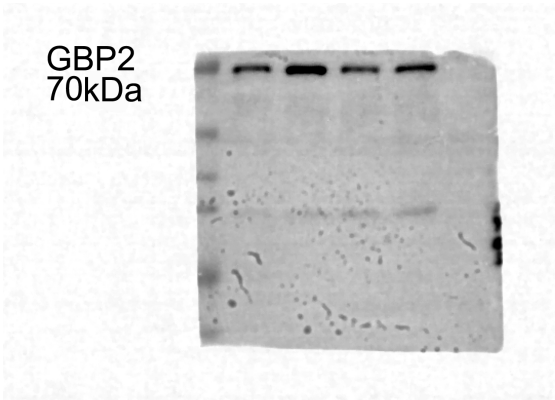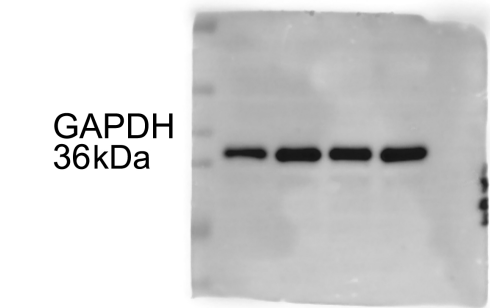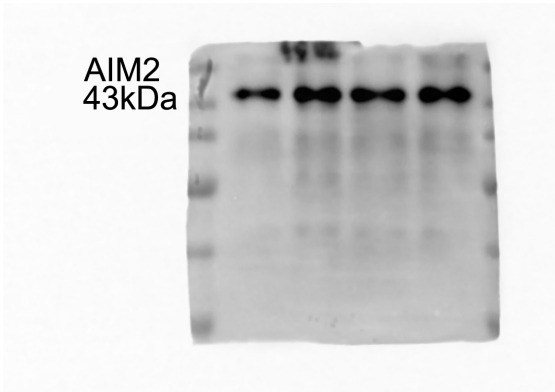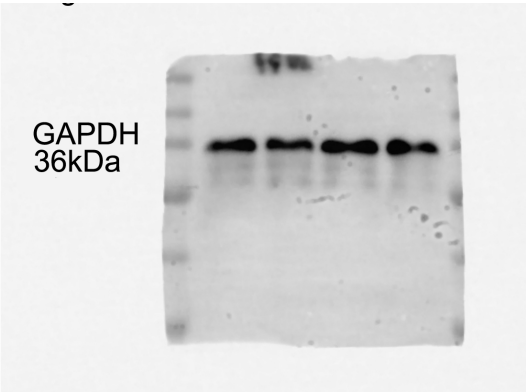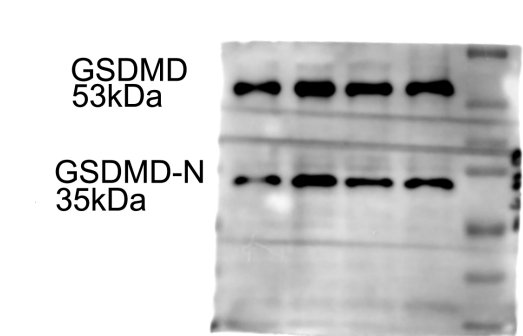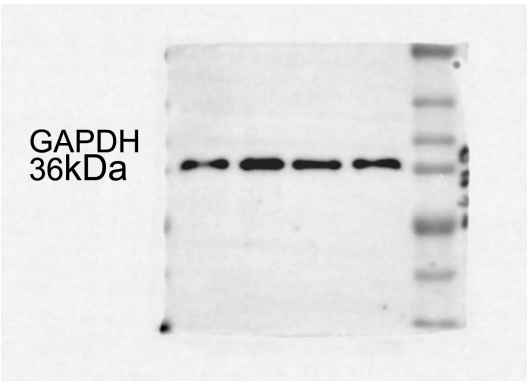

Supplement: S1 File — (PDF) [file pone.0344601.s001.pdf]
